# A novel *TRPS1* gene mutation causing trichorhinophalangeal syndrome with growth hormone responsive short stature: a case report and review of the literature

**DOI:** 10.1186/1687-9856-2014-16

**Published:** 2014-08-15

**Authors:** Lina Merjaneh, John S Parks, Andrew B Muir, Doris Fadoju

**Affiliations:** 1 Division of Endocrinology and Diabetes, Department of Pediatrics, Emory University School of Medicine, Atlanta, GA, USA

**Keywords:** Trichorhinophalangeal syndrome type I, Growth hormone, *TRPS1*, Conical epiphyses, Sparse eyebrows, Hip dysplasia, Missense mutation, Nonsense mutation

## Abstract

The role of growth hormone (GH) and its therapeutic supplementation in the trichorhinophalangeal syndrome type I (TRPS I) is not well delineated. TRPS I is a rare congenital syndrome, characterized by craniofacial and skeletal malformations including short stature, sparse, thin scalp hair and lateral eyebrows, pear-shaped nose, cone shaped epiphyses and hip dysplasia. It is inherited in an autosomal dominant manner and caused by haploinsufficiency of the *TRPS1* gene. We report a family (Mother and 3 of her 4 children) with a novel mutation in the *TRPS1* gene. The diagnosis was suspected only after meeting all family members and comparing affected and unaffected siblings since the features of this syndrome might be subtle. The eldest sibling, who had neither GH deficiency nor insensitivity, improved his growth velocity and height SDS after 2 years of treatment with exogenous GH. No change in growth velocity was observed in the untreated siblings during this same period. This report emphasizes the importance of examining all family members when suspecting a genetic syndrome. It also demonstrates the therapeutic effect of GH treatment in TRPS I despite normal GH-IGF1 axis. A review of the literature is included to address whether TRPS I is associated with: a) GH deficiency, b) GH resistance, or c) GH-responsive short stature. More studies are needed before recommending GH treatment for TRPS I but a trial should be considered on an individual basis.

## Background

Trichorhinophalangeal syndrome (TRPS) is a rare malformation syndrome characterized by distinctive craniofacial and skeletal abnormalities. It was first described by Klingmuller in 1956 [1] and then named the trichorhinophalangeal syndrome by Giedion in 1966 [2]. Three subtypes have been described. Features common to all subtypes are sparse scalp hair and lateral eyebrows, bulbous tip of the nose, long flat philtrum, thin upper vermilion border and protruding ears. Skeletal abnormalities include cone shaped epiphyses at the phalanges, hip dysplasia (resembling but not identical to Legg-Calve-Perthes disease) and short stature [3]. Patients with TRPS II have multiple cartilaginous exostoses in addition to the previous findings. The presence of severe brachydactyly and severe short stature with the absence of exostoses differentiates TRPS III.

TRPS is inherited in an autosomal dominant manner [4]. The *TRPS1* gene maps to 8q24 [5,6] and encodes a zinc finger transcription repressor involved in the regulation of chondrocyte and perichondrium development [7]. It has 2 potential nuclear localization signals and 9 different zinc-finger motifs [8,9]. Haploinsufficiency for this putative transcription factor causes TRPS I [9]. Most patients with nonsense mutations show the TRPS I phenotype, while patients with missense mutations in the GATA, DNA-binding, zinc-finger show the more severe TRPS III phenotype [10]. TRPS II is a contiguous gene deletion syndrome involving the *EXT1* and *TRPS1* genes resulting in the additional finding of multiple exostoses.

Responsiveness of growth to exogenous growth hormone (GH) in patients with TRPS I has been inconsistent according to previous reports. Naselli et al. and Sohn et al. reported failure of GH treatment in a pair of monozygotic twins and 2 other unrelated patients with TRPS I [11,12], whereas Stagi et al. and Sarafoglou et al. reported that GH treatment was effective in improving height velocity in 4 patients with TRPS I [13,14].

We report here a family with TRPS I with a novel nonsense mutation in the *TRPS1* gene. The mother and 3 of her 4 children have clinical features of TRPS I. The eldest sibling, who had normal GH secretion determined by provocative testing, a normal GH-IGF-1 axis, and normal bone mineral density (BMD), accelerated his linear growth velocity over a 2 year GH treatment period.

## Case presentation

The patient presented at age 7 years 2 months for evaluation of short stature. He was born at term by vaginal delivery with a birth weight of 3.373 kg. His history was significant for severe gastroesophageal reflux in infancy causing poor weight gain. He also suffered from recurrent ear infections. At the age of 2 years, he was diagnosed with atypical Legg-Calve-Perthes disease in the left hip that did not require casting or surgery. His height had been tracking below the first percentile and his weight between the 10–25^th^ percentile since age 3 years. Midparental height was 171 cm (25^th^ percentile) with a maternal height of 157.5 cm and paternal height of 172 cm.

**Figure 1:**
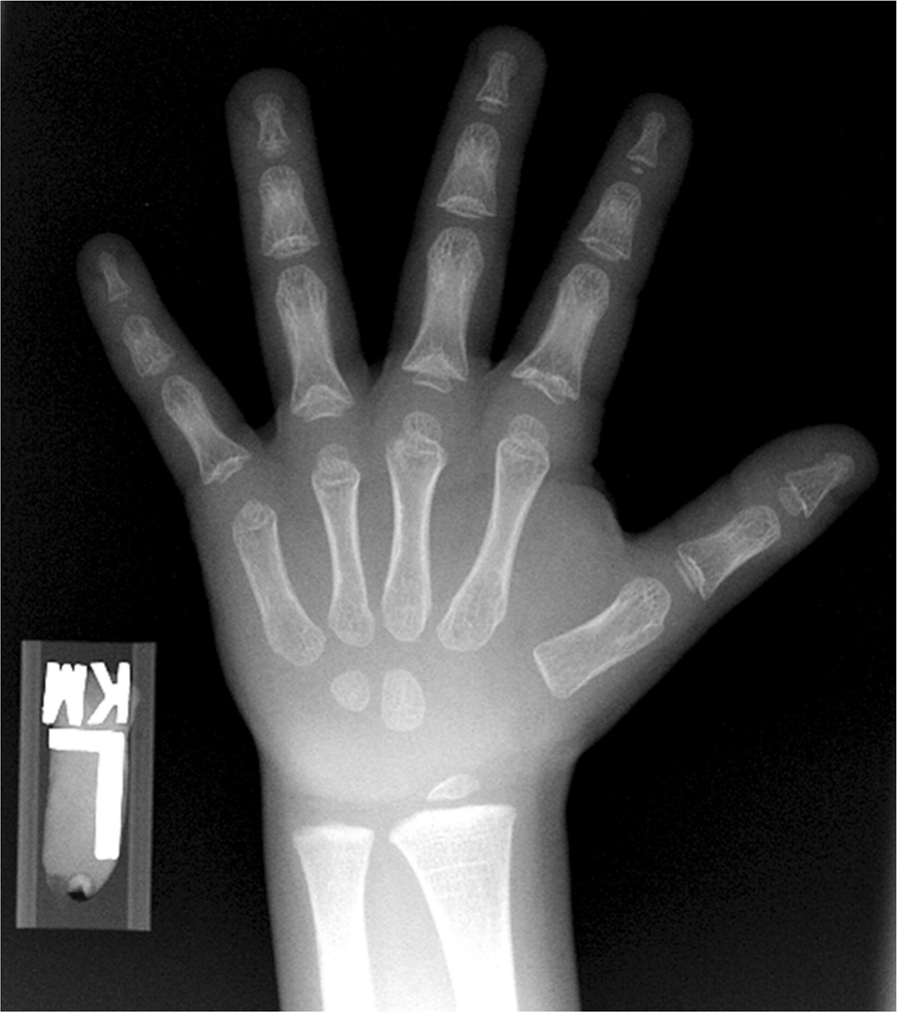
Bone age radiograph of patient’s younger brother showing the cone shaped epiphyses with diffuse shortening of the phalanges.

At the time of the initial evaluation, the patient’s height was 110.3 cm (1^st^ percentile, −2.25 SD) and weight was 22 kg (25–50^th^). Although no dysmorphia was noted at that visit, suspicion for TRPS I subsequently arose after other features of TRPS were identified in 2 affected siblings (younger brother and sister). The combined features of TRPS I seen in the mother and 3 of her children were: short stature, thin sparse scalp hair, long flat philtrum, thin upper vermilion border, bulbous nose tip, high arched narrow palate and overcrowded teeth. There was one unaffected younger brother.Initial laboratory evaluation (Quest Diagnostics, Atlanta, GA) showed the following: TSH 2.13 uIU/mL (nl: 0.5–4.5), Free T4 1.24 ng/dL (nl: 0.9–1.6), IGF1 180 ng/mL (nl: 37–217), IGFBP3 3.9 mg/L (nl: 1.4–6.1). Bone age radiograph revealed the presence of normally-shaped phalangeal epiphyses with a significantly delayed bone age of 3 years 6 months (−4.3 SD). However, bone age radiograph of his younger brother who was 5 years old revealed the presence of cone-shaped phalangeal epiphyses with diffuse shortening of the phalanges in addition to delayed bone age of 2–3 years (−3.4 SD) (Figure 1). Peak serum GH concentrations were 15.3 ng/mL after insulin and 8.2 ng/mL after arginine. Serum cortisol rose from 5.9 to 18.8 ug/dL as serum glucose dropped to 43 mg/dL after insulin. Dual-energy X-ray absorptiometry (DEXA) scan was normal with whole body BMD of 0.87 g/cm2 (Z score 0.6) and spinal BMD of 0.8 g/cm2 (Z score −0.1). Similarly, DEXA scan of the younger sibling showed normal whole body BMD of 0.78 g/cm2 (Z score 0.0) and borderline diminished spinal BMD of 0.5 g/cm2 (Z score −1.4).

Gene sequencing (Prevention Genetics, Marshfield, WI) of the patient and his younger affected brother demonstrated heterozygosity for a nonsense mutation, c.3368 G > A (p.Trp1123Stop).

**Figure 2:**
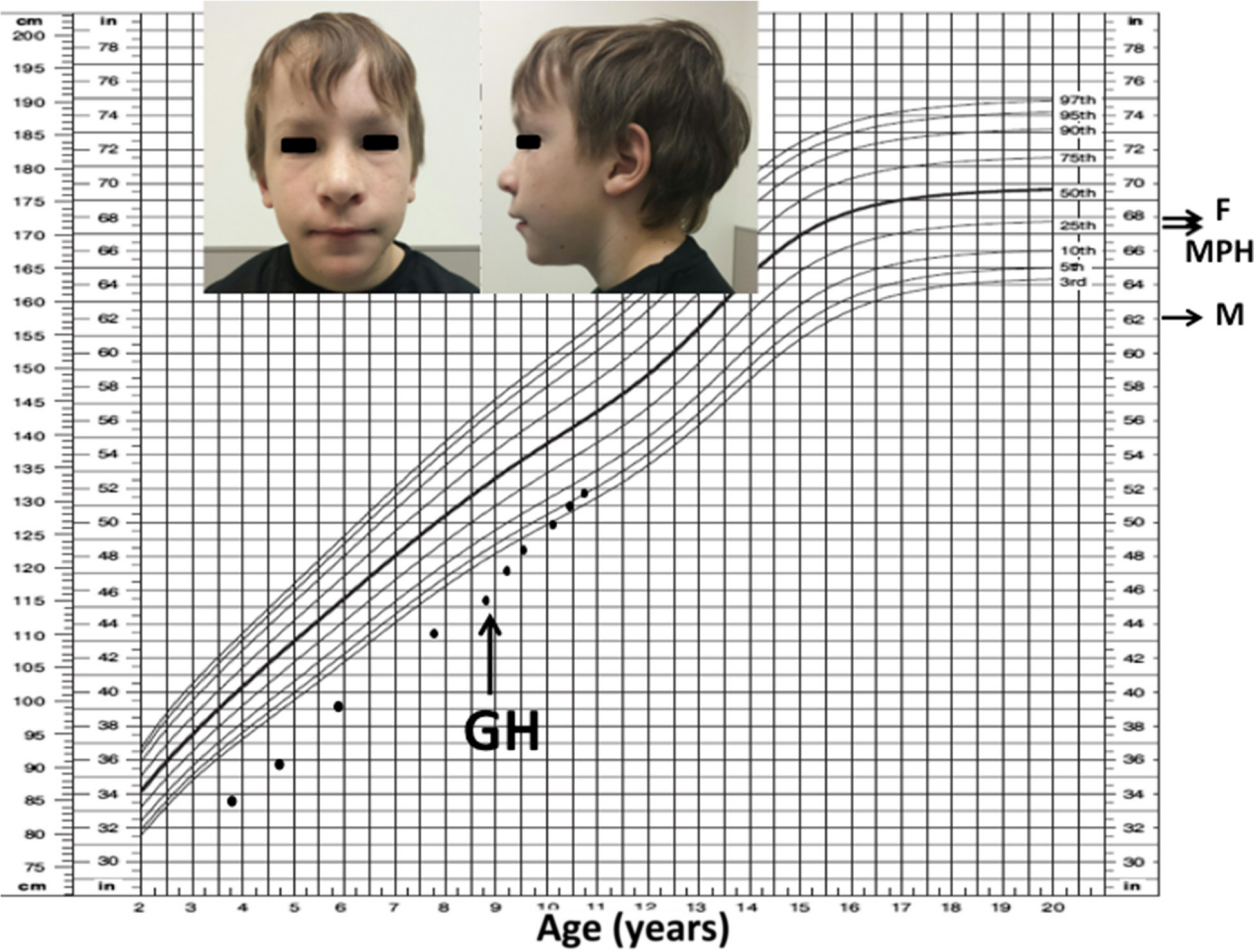
**Growth chart of patient before and after starting growth hormone.** F: father’s height, M: mother’s height. MPH: midparental height.

Daily subcutaneous GH was administered for 2 years beginning at age 8 years 4 months with a mean dose of 0.28 mg/kg/week (Table 1). The height SDS score increased from −2.4 to −1.4 and his growth velocity increased from 4.7 cm/year before treatment to 7.7 cm/year during treatment (Figure 2). Bone age advanced by 2.5 years (from 4.5 to 7 years) during 2 years of GH treatment. He remained prepubertal during treatment. In comparison, his younger brother whose application for GH treatment was denied remained below the 1^st^ percentile for height with a growth velocity of 5.5 cm/year during that time.

**Table 1:** Growth data and GH dose in our patient

**Chronological age (CA)**	**Height (cm)**	**Height Percentile/SDS**	**Growth velocity (cm/year)**	**Bone age (BA)**	**GH dose (mg/kg/week)**
7 y 2 m	110.3	1^st^/- 2.33		3 y 6 m	0
8 y 4 m	115.8	1^st^/- 2.38	4.7	4 y 6 m	Started at 0.29
9 y 4 m	123.8	3^rd^/- 1.85	8		0.28
10 y 4 m	131.2	9^th^/1.39	7.4	7 y	0.27

SDS: Standard deviation score.

## Discussion

TRPS I is an infrequently diagnosed cause of short stature that is associated with subtle dysmorphia. It displays clinical heterogeneity, even within a family. In the case reported here, although the diagnosis was apparent in retrospect, it was not until the findings of 3 affected siblings were combined that the diagnosis was made.

### Genetics and pathophysiology of TRPS I

Consistent with the TRPS I phenotype, cartilage and hair follicles are among the limited tissues that express the gene. It is postulated that TRPS1 (the protein product of *TRPS1*) deficiency impairs endochondral cartilage differentiation and epithelial/mesenchymal cell interactions in developing hair follicles [15]. Mechanistically, it is hypothesized that TRPS1 deficiency induces mitotic arrest in prometaphase cells as a result of abnormal chromatin condensation caused in part by excessive histone deacetylation [16]. Normally, TRPS1 homodimers complex with GATA binding protein sequences in DNA to repress transcription of target genes, an effect that is suppressed when TRPS1 is complexed with either the dynein light-chain LC8 or the ring finger protein RNF4.

Mutation analysis of *TRPS1* has revealed that missense mutations restricted to the GATA DNA-binding domains (aa 886–952) caused the more severe musculoskeletal anomalies associated with TRPS III. In 2 cases where missense mutations occurred outside the GATA zinc finger and in all reported nonsense single base pair mutations, the milder TRPS I phenotype arose [17] as in our case. The previously unreported mutation in this family is predicted to stop translation between the 7^th^ and 8^th^ zinc finger domains of TRPS1. In theory, translation of the nuclear translocation signal persists, but the mutation prevents extension of TRPS1 to the 2 Ikaros family zinc fingers at the protein’s carboxy terminus. These are necessary for repression of GATA-driven transcriptional signals [18]. Although the mutation also interrupts the RNF4 binding site between amino acids 985 – 1184, this change should be inconsequential in the absence of the Ikaros domains.

Ludecke et al. reported the average height of 75 patients with TRPS to be 1.41 SD below the average height of the respective population (SD = 1.15). Their data included children and adults with TRPS I and III [10]. Four previous reports of growth hormone treatment for short stature in patients with TRPS I have yielded conflicting results (Table 2). Naselli et al. first reported no growth promotion by GH in monozygotic twins with TRPS I [11]. Although the report indicated both children had GH deficiency, the actual GH stimulation tests results were not included. Prior to the start of growth hormone, both girls were prepubertal and had bone age delays of 2–3 years. Their treatment was discontinued after 12 months owing to its ineffectiveness.

**Table 2:** Summary of the reports on GH axis evaluation and treatment in patients with TRPS I

	**Patients (gender)**	**Genetic diagnosis**	**GH status**	**Chronological age at GH start**	**Bone age at GH start**	**Pubertal staging**	**GH dose mg/kgnwk**	**Height SDS change**
Naselli et al. 1998 [11]	Patient 1 (F)	Not reported	Deficient, based on GH stim test and IGF1 level, no data	11.1 y	8–9 y	Prepubertal	0.23	None
	Patient 2 (F)	Not reported	Deficient, based on GH stim test and IGF1 level, no data	11.1 y	8–9 y	Prepubertal	0.23	None
Stagi et al. 2008 [13]	Patient 3 (M)	Not reported	Partial deficiency, Peak after arginine 6.8, after insulin 12.7, low nocturnal GH 2.7	12 y	9 y 6 m	G2, PH2, T4 ml bilaterally	0.26	+ 0.7 SDS over 5 years
	Patient 4 (M)	c2722C > T (p.R908X)	Partial deficiency. Peak after Clonidine 10.2, peak after insulin5.4, low nocturnal GH 2.38	9 y 9 m	7 y8 m	Prepubertal	0.26	+ 1.9 SDS over 7 years
Sarafoglou et al. 2010 [14]	Patient 5 (M)	Not reported	No deficiency. Low IGF1 and normal IGFBP3	7 y	~3 y delay	Prepubertal	0.3–0.43	+1.81 SDS over 3 years
	Patient 6 (F)	Not reported	No deficiency. Low IGF1 and normal IGFBP3	6.95 y	~ 6 m delay	Prepubertal	0.34–0.54	+1.95 SDS over 2 years
Sohn et al. 2012 [12]	Patient 7 (F)	c2520dupT (p.Arg841LysfsX3)	Deficient (peak after insulin 3.17 and after L-dopa 5)	4 y	2.6 y	Prepubertal	0.2	+ 0.4 SD over 10 years
	Patient 8 (M)	c1630C > T (p.Arg544X)	Not deficient (peak after glucagon 9.86, after L-dopa 9.7)	14 y	16 y	Pubertal	Not reported	None (1 cm over 6 months)

Sohn et al. also reported 2 Korean patients with TRPS I who were unresponsive to GH treatment [12]. One was a 4 year old girl who was GH deficient based on stimulation testing. Despite a 10 year course of GH (0.2 mg/kg/week), her height SDS response to treatment was not impressive as she only gained 0.4 SDS change in her height over that time. The other was a 14 year old boy with a bone age of 16 years and a peak circulating GH concentration of 9.9 ng/mL after receiving glucagon. He did not show any growth velocity response after a 6 month GH trial, a circumstance that is expected with his advanced bone age of 16 years.

Stagi et al. [13] described 2 GH-responsive patients with TRPS I who had normal GH responses to provocative testing [13]. The authors however considered the patients to both have partial growth hormone deficiency based on low nocturnal mean GH concentrations. One had a height SDS of −2.2 and a bone age of 9.5 years at age 12 years. After 5 years of GH treatment, his adult height was −1.5 SDS, well above his mid-parental target height of −3.5 SDS. The other patient had a height SDS of −2.0 and bone age of 7 years 8 months at age 9 years 9 months. His growth velocity doubled during the first year of treatment and his final height was −0.1 SDS. The rate of bone age progression during GH treatment was not reported for either patient and unfortunately, neither experienced improvements of their pre-treatment osteopenia.

Sarafoglou et al. [14] described accelerated growth and improved bone mineral density after GH treatment in 2 siblings with TRPS I [14]. A 6.3 year old male had a height SDS of −2.7, weight SDS −3.6, pre-treatment growth velocity of 2.5 cm/year, and bone age of 3.5 years. In the face of normal GH provocative testing, his serum IGF-1 concentration of 81 ng/mL was considered to indicate GH resistance, notwithstanding an IGFBP3 concentration of 1.7 mcg/mL. Growth hormone (0.3 – 0.43 mg/kg/week) for 3.2 years resulted in an increased height SDS to −1.37, weight SDS −1.06, mean growth velocity of 6.7 cm/year and bone age progression to 8 years. This patient’s sister had a height SDS of −2.76, weight SDS of −3.35 at 6.3 years, pre-treatment growth velocity of 3.9 cm/year, and bone age of 5.75 years. She too was designated as GH resistant on the basis of normal provocative GH testing, serum IGF1 concentration of 81 ng/mL, and serum IGFBP3 concentration of 3.5 mg/L. Over 3 years of GH treatment, her height SDS rose to −1.1 and her bone age increased to 7.8 years. The IGF1 concentrations of both children rose by 2 to 5 fold over their baseline levels while they received exogenous GH doses of 0.3 to 0.5 mg/kg/week. Improved BMD was reported with GH treatment, but only in the female patient.

Combined with the family reported here, the literature allows a series of questions to be addressed.

### Is short stature in TRPS I associated with GH deficiency?

The normal responses of growth hormone to insulin and arginine in the child reported here are consistent with previous reports that suggest classic GH deficiency is not common among patients with TRPS I. Of the 6 published patients who underwent provocative GH testing, 5 had normal responses and one had a peak response of 5 ng/mL after L-dopa administration. The inability of provocative GH testing to discern normal from pathological states when the peak response falls above 2.5 ng/mL is well described [19,20]. Stagi et al. diagnosed partial GH deficiency in their patients based on serial measurements of nocturnal serum growth hormone concentrations. The reliability of this procedure is questionable since its diagnostic usefulness was reported to be inferior to stimulation testing [21].

### Is TRPS I associated with GH resistance?

Although Sarafoglou et al. claimed their patients both had GH resistance, the baseline serum IGF1 SDS scores were −1.5 in both patients and these were accompanied by normal IGFBP3 concentrations. With weight SDS scores significantly below the height SDS scores, one should consider the possibility that malnutrition in the baseline state may have contributed to selective depressions of IGF-1. Nutritional counseling was not addressed in the report. Finally, the marked increase of circulating IGF1 after exogenous GH administration is inconsistent with a GH resistant state. The normal pre-treatment serum IGF1 and IGFBP3 concentrations in the children reported here and their responses to exogenous GH administration are also evidence against a growth hormone resistant state.

### Is short stature in TRPS I GH responsive?

Increased growth velocity and final height after GH treatment are well described in short children with normal function of their GH-IGF1 axis. The patient reported here increased his growth velocity by 63 percent over the 2 years of treatment. This compares to a mean change in growth velocity of 66–168 percent among the responding patients in the previously published reports. The previous reports may indicate that the early initiation of GH treatment is associated with better height outcomes. Similar observations were reported after GH treatment in children with GH-sufficient, idiopathic short stature [22]. Sohn et al. however reported suboptimal growth in a girl with TRPS I, despite starting a 10 year course of GH supplementation at 4 years of age [12]. Factors contributing to her poor response may include the low GH dose (0.2 mg/kg/week), her midparental height, and the adherence to her recommended treatment. A dose dependent effect may be reflected in better outcomes reported by Sarafoglou et al. [14] in 2 children receiving GH doses of 0.3 to 0.5 mg/kg/week.

The mechanism by which growth hormone therapy could accelerate linear growth in children with TRPS I is unknown and could be complex. In a cell culture model that may mimic TRPS1 mutations however, IGF-1 expression by a chondrogenic cell line derived from a murine teratoma (ATDC5) was reduced by blockade of TRPS1 expression with microRNA [23]. It is therefore possible that high systemic IGF-1 concentrations, resulting from growth hormone therapy, compensate for low local IGF-1 concentrations in the growth plates of individuals with TRPS1 mutations.

These cases also incidentally indicate the need to consider whether routine evaluation of bone mineral density is indicated in patients with TRPS I and whether it may be increased by GH treatment.

## Conclusions

We report here a family with TRPS I (the mother and 3 of her 4 children) caused by a novel mutation in the *TRPS1* gene. The older child was treated with growth hormone for 2 years using a dose that ranged from 0.24 to 0.32 mg/kg/week. Despite the fact that he did not have growth hormone deficiency or insensitivity, he showed an excellent response to treatment with +1 SDS gain in his height over 2 years.

At a time when GH treatment is becoming more difficult to provide due to restrictions applied by insurance companies, this report supports the consideration of GH treatment for patients affected with TRPS I. Short stature could be a contributing risk factor for psychosocial problems in some children, such as social immaturity, low self-esteem, and being bullied in addition to possible disadvantages in terms of obtaining employment and finding partners as adults [24-26]. More data are needed before TRPS I can be considered an indication for GH treatment, but our case proves that GH improves height outcome in TRPS I in the short term. Effect on final adult height will need longer follow-up of this patient until adulthood. The appropriate advancement of bone age during treatment suggests that his final height will not be adversely affected with GH treatment.

## Consent

Written informed consent was obtained from the patient’s mother for publication of this Case report and any accompanying images. A copy of the written consent is available for review by the Editor-in-Chief of this journal.

## Abbreviations

TRPS: Trichorhinophalangeal syndrome; GH: Growth hormone; DEXA: Dual-energy X-ray absorptiometry; BMD: Bone mineral density.

## Competing interests

The authors declare no competing interests.

## Authors’ contributions

LM drafted the initial manuscript, and approved the final manuscript as submitted. JSP, AM and DF reviewed/edited the manuscript and approved the final manuscript as submitted. All authors read and approved the final manuscript.
